# Surgery in Mercy Hospital

**Published:** 1881-01

**Authors:** J. J. Larkin

**Affiliations:** Former House Surgeon, Mercy Hospital, Chicago; South Chicago; Cor. Commercial Ave. and 92d St.


### Clinical Reports.

### NOTES FROM HOSPITAL PRACTICE.

### ARTICLE VIII.

*Surgery in Mercy Hospital.*

In presenting this incomplete sketch of surgical work in Mercy Hospital, I purpose speaking of the general management of cases, number and character of operations, and of results, hoping to give such facts as will interest and instruct the reader.

Too many reports that find their way into medical journals are composed of glittering generalities, sparkling rhetorical figures that illuminate the *successes* while they obscure the instructive *failures.* The data for this report may be found in the daily Surgical Records of the Hospital from October 1, 1879, to April 1, 1880.

Surgical patients have "*the best the house affords*," of large, well-lighted, well-ventilated wards, and equally well-lighted, and ventilated elegant private rooms. Especial attention is given to heating and ventilating all apartments.

Dr. Edmund Andrews, Prof. of Principles and Practice of Surgery in Chicago Medical College, is Surgeon-in-Chief, the House Surgeon acting entirely under his orders. Each patient has his own sponges, towels, wash-bowls and bandages; in no case is he allowed to use his neighbor's. All wounds are dressed by the house surgeon or his assistants, who are members of the *middle* or *senior* classes at the Chicago Medical College. Nurses are not allowed to do dressing.

Wounds are dressed *once* or twice a day, as the case demands, and treated in the following way: first, they are thoroughly cleaned with tepid water containing two and a half per cent. carbolic acid, then covered with cotton saturated with olive oil containing eight per cent. carbolic acid; this is held in place by several turns of a roller bandage.

After dressing each patient the dresser disinfects his hands and instruments that the next patient may not be contaminated. The house surgeon makes a careful record of each patient's condition at least twice a day. Sulph. cinchonidia, quinine and the preparations of iron are used for general tonic purposes; quinine, aconite and salicylic acid are employed to control temperature and pulse.

In all operative cases, when it is possible, a preparatory course of treatment for a few days is employed, that the system may be in the best possible condition for operation. Dr. Andrews performs all operations in the Hospital amphitheater, in the presence of the class from the college. Ether is the anæsthetic employed. The result of some experiments with ether will appear later in this report.

During the six months previously mentioned there were 163 surgical cases treated in Mercy Hospital; 125 operations performed; 75 of these under an anæsthetic. Among some of the most important cases were 20 of fractures, 10 necroses, 2 stone in bladder, 6 epithelioma of lip, 5 hare-lip and cleft palate, 13 cancerous and fatty growths, 2 abscess of tibia, 3 aneurisms, 2 extrophy of bladder, 2 amputations and 1 tracheotomy, performed.

The average time that fracture cases remained in the Hospital was 27 days, necrosis cases remained 47 days, amputations 23 days, staphylorraphy cases 15 days. Abscess of tibia 21 days, epithelioma of lip 12 days, and removal of other growths 14 days.

Of the whole number of cases treated 78 were discharged cured, 63 improved, 16 unimproved, and 6 died. Showing that *about* three and a half per cent. of all proved fatal, ten per cent. unimproved, thirty-nine per cent. improved, forty-nine per cent. cured.

It being thought that a volatile oil mixed with ether would lessen the tendency to vomit, we tried the use of ℥j of oil of turpentine to 1 pound can of ether, in 74 cases of which 21 vomited.

Twenty-eight cases were tried without the turpentine, 6 vomited. About twenty-eight per cent. of the former and twenty-two per cent. of the latter vomited. We stopped the use of the turpentine. (Twenty-five of the above cases were in Dr. Andrews' private practice.)

A report such as this must ever be incomplete and fragmentary, but if any information can be derived from it I shall be well pleased. As I previously remarked, every statement here is based on the surgical records of the Hospital, and can be verified by reference to them, now in possession of Dr. Edmund Andrews.

J. J. LARKIN, M.D.,
*Former House Surgeon, Mercy Hospital, Chicago.*
SOUTH CHICAGO, Dec. 15, 1880.
Cor. Commercial Ave. and 92d St.

